# Effect of intracerebroventricular injection of GABA receptors antagonists on morphine-induced changes in GABA and GLU transmission within the mPFC: an *in vivo* microdialysis study

**DOI:** 10.22038/ijbms.2019.28478.6925

**Published:** 2019-03

**Authors:** Effat Ramshini, Hojjatallah Alaei, Parham Reisi, Naser Naghdi, Hossein Afrozi, Samaneh Alaei, Maryam Alehashem, Shahrzad Eftekharvaghefi

**Affiliations:** 1 Department of Physiology, School of Medicine, Isfahan University of Medical Sciences, Isfahan, Iran; 2 Department of Physiology, Pasteur Institute of Iran, Tehran, Iran; 3 Drug Research Center of Daropakhsh, Tehran, Iran; 4 Tracheal Diseases Research Center, National Research Institute of Tuberculosis and Lung Diseases (NRITLD), Shahid Beheshti University of Medical Sciences, Tehran, Iran; 5 Department of Physiology, Kerman University of Medical Sciences, Kerman, Iran

**Keywords:** Addiction, GABA-A receptor-antagonists, GABA-B receptor-antagonists, Morphine, Prefrontal cortex

## Abstract

**Objective(s):**

Many studies have focused on ventral tegmental area than of other mesocorticolimbic areas, and implicated a key role for the medial prefrontal cortex (mPFC) in the development of addictive behaviors. So far, the role of gamma-aminobutyric acid (GABA) receptors in the discriminative properties of morphine has received little attention and few studies evaluated the role of these receptors in drug dependence. Hence, we investigated the role of this receptor on morphine- induced GABA/ glutamate (GLU) changes in the mPFC following morphine administration using *in vivo* microdialysis.

**Materials and Methods:**

In this study, 60 rats weighing 270-300 g were divided into six groups. First, microdialysis probe was inserted into the mPFC and was perfused with artificial cerebrospinal fluid and collected the baseline samples in all groups. In saline and morphine groups, the saline, in phaclophen and (phaclofen+morphine) groups, phaclofen (100 nmol), and in bicuculline and (bicuculline+morphine) groups, bicuculline (20 nmol) was injected intracerebroventricular. In saline, phaclofen and bicuculline groups 20 min later, animals received saline (0.2 ml, IP) and others groups received morphine (20 mg/kg, IP).

**Results:**

Our results showed that morphine increased the average concentration of GABA and decreased the concentration of GLU within mPFC. Pretreatment with phaclofen and bicuculline 20 min before morphine administration had no effect on GABA and GLU release for 100 min.

**Conclusion:**

The present study indicated that morphine influence the GABA and GLU transmission in mPFC. Therefore evaluation of neurochemistry changes of this neural circuitry may provide further insight into the mechanisms underlying drug dependence.

## Introduction

Repeated exposure to morphine is often accompanied with dependence (1). Most available treatments are still relatively ineffective because the underlying mechanisms are not completely understood (2). In order to develop new pharmacothraputic treatments for recovery of drug dependence, it is necessary to gain insight into the neuronal mechanisms underlying the addiction (3, 4). Microdialysis can be considered a “classical technique” for investigation of biochemical events in the extracellular fluid of any tissue. The mesocorticolimbic system is one of the brain regions involved in reward circuit, which are consisted of ventral tegmental area (VTA), nucleus accumbens (NAc) and the medial prefrontal cortex (mPFC) (5-8) .Behavioral studies demonstrated the key role of the medial prefrontal cortex in the drug-reinforced behavior. Taken together, these neuroanatomical and neurochemical communications in the mPFC suggest direct and indirect functions of morphine in this area (9). Medial prefrontal cortex receives dense dopamine (DA) innervations from the VTA and sends glutamatergic afferents to the VTA and NAc (10, 11) . The two major cell types that exist in the mPFC are glutamate (GLU)-containing pyramidal projection neurons and gamma-aminobutyric acid (GABA) local interneurons (12). Although most studies have focused on the VTA rather than other mesocorticolimbic areas, several studies have implicated a key role for the mPFC in the development of addictive behaviors (10). Previous studies indicated that the PFC receives both dopamine and GABA projection from the ventral tegmental area and GLU -containing pyramidal neurons in the medial prefrontal cortex project to the VTA where they synapse on mesocorticolimbic GABA interneurons (13).

A growing body of evidence indicated that the alterations of multiple neurotransmitters including dopamine, serotonin, GLU and GABA contribute to the drug dependence (10). GABA is the major inhibitory neurotransmitter in CNS and is involved in morphine dependence (14). The two major GABA receptor subtypes, the inotropic GABAA receptors and the metabotropic GABAB receptors subtype, are abundantly distributed throughout the cortex (15). Presynaptic GABAB receptors seem to modulate the release of several neurotransmitters (16, 17).So far the role of GABA receptors in the discriminative properties of morphine has received little attention and few studies evaluated the role of these receptors in drug dependence (18). Numerous studies found that morphine can modulate neurotransmitters activity and indicated that the GABAergic and glutamatergic outputs from the mPFC play a critical role in the development of drug dependence (19); hence, understanding the mechanism of action of morphine on GABAergic and glutamatergic neurotransmission may elucidate its role in the development of morphine-induced reinforcement (20). 

Thus, the present study was undertaken to evaluate the neuropharmacological effects of GABA receptors on extracellular GABA and GLU levels within the mPFC in response to a single dose of morphine using *in vivo* microdialysis.

## Material and methods

***Animals*** ***and experimental groups***

In this study, 60 male wistar rats weighing 270-300 g were divided into six groups: Saline, phaclophen, bicuculline, Morphine, phaclofen+morphine and bicuculline+morphine groups. The animals were housed in standard plastic laboratory cages in a temperature-controlled (22±1 ^°^C) colony room that was maintained on a standard 12 hr light/12 hr dark cycle with food and water freely available. All animals were allowed to adapt to the laboratory conditions for at least 1 week before experiments and were handled for 5 min per day during this adaptation period. All experiments were conducted during the light phase. All procedures in this study were approved and reviewed by the Ethic Committee for Animal Experiments at Isfahan University of Medical Sciences. Experimental procedures and care of the animals were in accordance with the Guide for the Care and Use of Laboratory Animals, Eighth Edition, (2011, published by The National Academies Press, 2101 Constitution Ave. NW, Washington, DC 20055, USA). 

Saline group: animals received saline (5 µl, ICV), after 20 min received saline (0.2 ml IP)

Phaclofen group: animals received GABAB receptors antagonist (phaclofen, 100 nmol, ICV), after 20 min received saline (0.2 ml IP) 

Bicuculline groups: animals received GABAA receptors antagonist (bicuculline, 20 nmol, ICV), after 20 min received saline (0.2 ml IP)

Morphine group: animals received saline (5 µl, ICV) and after 20 min received morphine (20 mg/kg IP) 

(Phaclofen+morphine) group: animals received GABAB receptors antagonist (phaclofen, 100 nmol, ICV), after 20 min received morphine (20 mg/kg, IP) 

(Bicuculline+morphine) groups: animals received GABAA receptors antagonist (bicuculline, 20 nmol, ICV), after 20 min received morphine (20 mg/kg, IP) 

***Drugs***

Morphine hydrochloride (Temad, Iran, 20 mg/kg, IP) (21), chloral hydrate (Merck, Germany, 350 mg/kg, IP) (22), phaclofen (Sigma-Aldrich, 100 nmol, ICV) (23) and bicuculline (Sigma-Aldrich, 20 nmol, ICV) (24) dissolved in saline 0.9%. Fresh solutions of all drugs were prepared prior to the experiments. Phaclofen and bicuculline were infused intracerebroventricular in a volume 5 µl via cannula that has been previously implanted in the cerebroventricular (CV) region and one end that was attached to a 50 µl Hamilton microsyringe. Solutions were infused slowly over a period of 5 min in each rat and cannula was kept in place for an additional 3 min to minimize the drug backflow into the injection track.

***Surgical procedures***

Rats were divided into six groups in this study (n=10 rats in each group) and were anesthetized with chloral hydrate (350 mg/kg, IP) and followed by if necessary maintenance doses. Animal placed in a stereotaxic frame in a flat-skull position and fixed. A midline incision was made on the skin and the underlying periosteum was retracted and the skull was cleaned. Then, we determined the mPFC and CV regions on the surface of skull by Paxinos and Watson atlas (mPFC: AP=+3 relative to bregma L=+0.6 relative to midline V=-2.5 relative to skull surface, CV: AP=0.8 relative to bregma, L=-1.5 relative to midline, V=-4.2 relative to skull surface) (25). First, a cannula was implanted in the CV region for injection of antagonists and fixed with dental cement. In the next stage, microdialysis probe by 23-gauge guide cannula was inserted in the mPFC via stereotaxic apparatus. The guide cannula was placed 1 mm above the intended site according to the Paxinos and Watson. In order to minimize trauma to brain tissue, the microdialysis probe lowered very slowly (0.2 mm/min).

**Figure 1:**
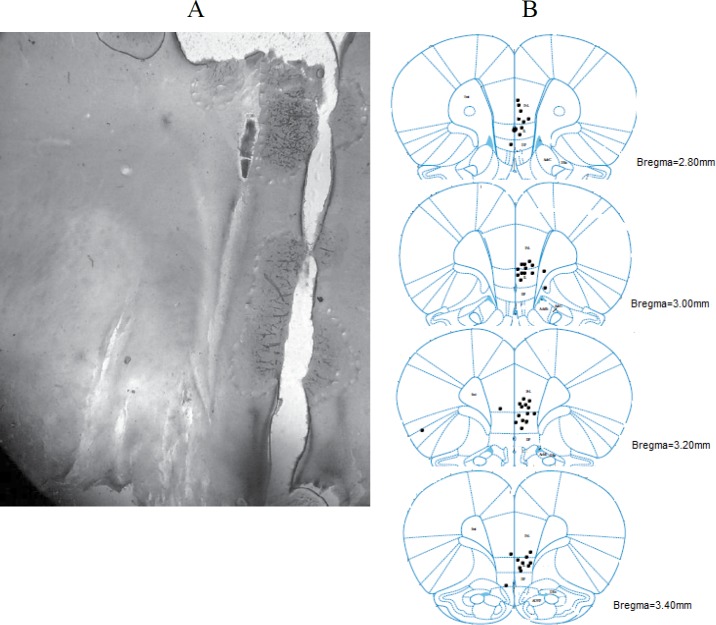
Schematic coronal section of rat brain adapted from an atlas (Paxinos and Watson, 2005). (A) Photomicrograph scan of a coronal section (50 μm) showing the probe site in the medial prefrontal cortex (mPFC). (B) The animals included in all groups equally distributed regarding to the place where the probe was implanted. The placements of probes implanted in the mPFC of rats included in the statistical analysis

**Figure 2:**
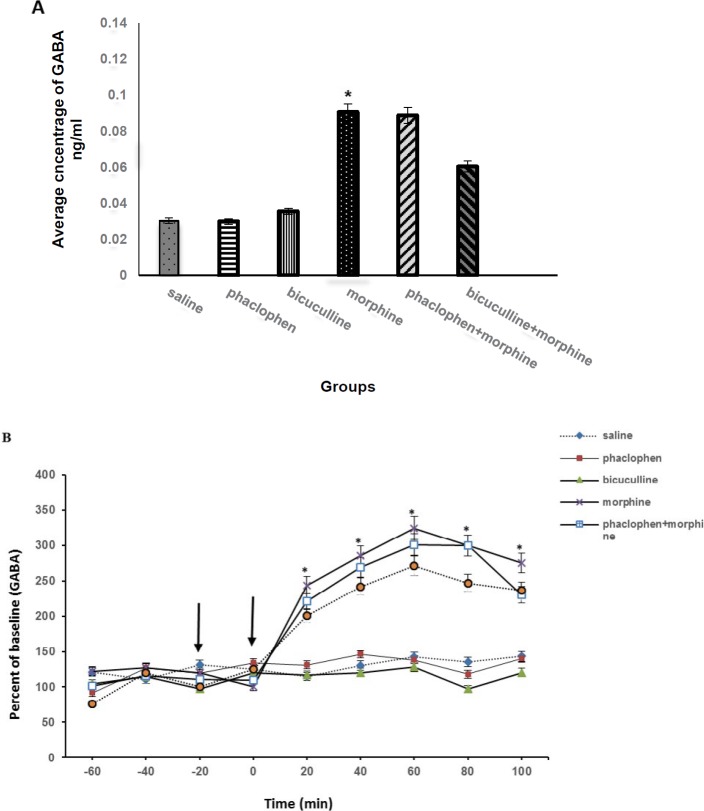
A) Effect of intracerebroventricular infusion of gamma-aminobutyric acid (GABA) receptors antagonists on the average concentration of GABA in medial prefrontal cortex (mPFC) following intraperitoneal morphine administration. Administration of morphine (20 mg/kg, IP) significantly increased the average concentration of GABA in comparison with saline group (*P<0.05). Pretreatment with Phaclofen (100 nmol, ICV) and bicuculline (20 nmol, ICV) 20 min before morphine administration had no effect on the concentration of GABA increased by morphine (*P<0.05). Also, administration of the same doses of phaclofen and bicuculline alone had no effect on the average concentration of GABA. Data are expressed as mean±SEM (n=10 rats in each group). B) Effect of intracerebroventricular infusion of GABA receptors antagonists on GABA release in mPFC following intraperitoneal morphine administration. Administration of morphine (20 mg/kg, IP) (second arrow) significantly increased GABA release in comparison with saline group (*P<0.05). Pretreatment with Phaclofen (100 nmol, ICV) and bicuculline (20 nmol, ICV) 20 min before morphine administration (first arrow) had no effect on GABA release during 100 min. Also, administration of the same doses of phaclofen and bicuculline alone had no effect on GABA release. Data are expressed as mean±SEM (n=10 rats in each group)

**Figure 3:**
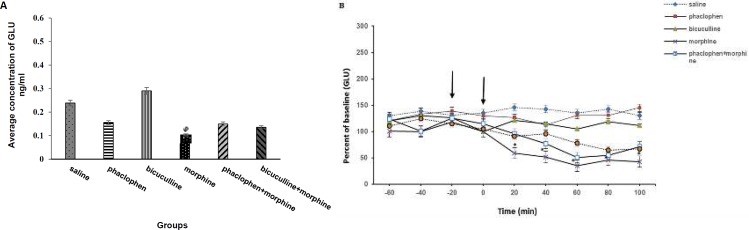
A) Effect of intracerebroventricular infusion of GABA receptors antagonists on the average concentration of GLU in mPFC following intraperitoneal morphine administration. Administration of morphine (20 mg/kg, IP) significantly decreased the average concentration of GLU in mPFC in comparative with saline group. Pretreatment with Phaclofen (100 nmol, ICV) and bicuculline (20 nmol, ICV) 20 min before morphine administration had no effect on concentration of GABA increased by morphine. Also, administration of the same doses of phaclophen and bicuculline alone had no effect on the average concentration of GLU (*P<0.05). Data are expressed as mean±SEM (n=10 rats in each group). B) Effect of intracerebroventricular infusion of GABA receptors antagonists on GLU release in mPFC following intraperitoneal morphine administration. Administration of morphine (20 mg/kg, IP) (second arrow) significantly decreased GLU release in comparative with saline group (*P<0.05). Pretreatment with phaclofen (100 nmol, ICV) and bicuculline (20 nmol, ICV) 20 min before morphine administration (first arrow) had no effect on GABA release during 100 min. Also, administration of the same doses of phaclofen and bicuculline alone had no effect on GLU release. Data are expressed as mean±SEM (n=10 rats in each group)

***In vivo microdialysis***

Microdialysis probe was connected to the microdialysis pump and perfused with artificial cerebrospinal fluid (aCSF: NaCl 145 mM/l KCl 2.7 mM/l CaCl_2_ (2H_2_O) 1.2 Mm/l MgCl_2_ (6H2O) 1 Mm/l Na_2_HPO_4_ (12H_2_O) 2 Mm/l PH= 7.4) at the flow rate of 2 µl/min for 60 min (21, 26). After 1 hr washing out period of mPFC region, dialysis samples were collected for 20 min periods successively in vials containing 10 µl of 0.5 M acetic acid to minimize decomposition. Up to four baseline samples were collected prior to treatment. Each lysate was measured independently, and the average of the measurements was shown. All CSF samples were kept on ice during collection and immediately stored at a temperature of minus 70 degrees for determination of basal GABA and GLU level via hyper pressure liquid chromatography-fluorescence detection (HPLC-FLD). The level of GABA and GLU measured by HPLC (column: C18- [150 mm×4.6 mm)-3 μm (phenomena Luna C18 (2) coupled to fluorescence detector (Ultrafluor -Lab Alliance): Ex=338 nm, Em=454 nm, PUMP: 515WATERS AND injector: Rheodyne-7725i, following pre-column derivatization with o-phthaladialdehyde (OPA). The mobile phase consisted of 0.05 M sodium acetate, tetrahydrofuran and methanol (50: 1: 49, v/v) adjusted to pH: 4. The mobile phase was filtered through Millipore 0.45 μm Durapore membrane filters and vacuum degassed prior to use. Chromatographic analyses were performed at 25±2 ^°^C. Compounds were eluted isocratically over 9 min running time at a flow rate of 1 ml/min (26-28). 

***Histology***

For the histological examination of microdialysis probe placement in mPFC, the animal was sacriﬁced by a high dose of the anesthetic then transcardially perfused with 100 ml of saline 0.9% followed by 100 ml of formalin 10%. The brain was removed and stored in 10% formalin for at least 72 hr. Brains were sectioned coronally at 50 μm by a freezing microtome (Leica, Germany) then were mounted on gelatin-coated slides and stained with cresyl violet. The track left by the probe was examined using a light microscope and their exact positions were determined by reference to a rat brain atlas (25). Only the dialysates of animals whose probes were implanted in the mPFC were included in the statistical analysis. (Figure 1). 

***Statistical analysis***

All given values are expressed as the percentage of the average of five baseline samples. The average concentration of five stable baseline samples was set at 100%. Statistical analysis was performed using repeated measures one-way analysis of variance (ANOVA), and Tukey test for *post-hoc* comparisons was performed when appropriate. All results were expressed as means±SEM. Differences with *P*<0.05 were considered significant. 

## Results

The current study evaluated the effects intracerebroventricular infusion of GABA receptors antagonists on GABA and GLU level in mPFC in animals that exposed to a single dose of morphine. Figure (2A) shows that administration of morphine (20 mg/kg, IP) significantly increased the average concentration of GABA within mPFC region in comparison with saline group. Pretreatment with Phaclofen (100 nmol, ICV) and bicuculline (20 nmol, ICV) 20 min before morphine administration had no effect on the concentration of morphine-induced GABA increase. Also, administration of same doses of phaclophen or bicuculline alone had no effect on the average concentration of GABA. Figure (2B) shows that GABA release increased 20 min after morphine administration and continues for 100 min. Maximum of GABA released in the 60^th ^min. Pretreatment with Phaclofen and bicuculline 20 min before morphine administration had no effect on GABA release during 100 min. Also, administration of the same doses of phaclophen and bicuculline alone had no effect on GABA release. 

Also, we evaluated the effects intracerebroventricular infusion of GABA receptors antagonists on GLU level in mPFC in animals that exposure to the single dose of morphine. Figure (3A) shows that administration of morphine (20 mg/kg, IP) significantly decreased the average concentration of GLU within mPFC region in comparative with saline group. Pretreatment with Phaclofen (100 nmol, ICV) and bicuculline (20 nmol, ICV) 20 min before morphine administration had no effect on the concentration of GLU decreased by morphine. Administration of the same doses of phaclophen or bicuculline alone had no effect on the average concentration of GLU. Figure (3B) shows that GLU release decreased 20 min after morphine administration and continues for 100 min. Minimum of GLU release occurring in the 60th min. Pretreatment with Phaclofen and bicuculline 20 min before morphine administration had no effect on GLU release during 100 min. Administration of the same doses of phaclophen and bicuculline alone had no effect on GLU release.

## Discussion

In the present study, we evaluated the concentration of GABA and GLU in the mPFC following morphine administration in the rat. Our results showed that administration of morphine not only increased GABA level but also decreased GLU level in mPFC. Our results appear to consist with some previous findings that described, systemic administration of morphine increase the level of GABA and decrease the level of GLU in the anterior cingulate cortex in the rat (29). Local perfusion of morphine into NAc dose-dependently increased GABA level, while attenuated GLU level in the NAc (30). Our results appear to contrast with some previous findings that showed, morphine administration reduced ventral pallidal GABA efflux (31) and increased GLU level in the locus coeruleus (LC) (32). It has been suggested that GABAergic and opioidergic systems are interconnected through μ-opioid receptors (4). It is possible that activation of GABA receptors is similar to mu opioid receptors and the receptors are indirectly activated by morphine on the GABAergic afferents. Morphine-induced activation of opioid receptors enhances GABAergic neuronal activities in the PFC, which may provide inhibitory influences on the glutamatergic pyramidal neurons (33). Exposure to morphine disinhibits GABAergic interneurons in the VTA and results in the increase of dopamine release into the PFC. This process could through activating GABAergic neurons directly or indirectly lead to inhibition of glutamatergic neurons in the PFC. In addition, morphine-induced modulation of dopaminergic projections from VTA and other brain regions to the mPFC could influence the metabolism and concentrations of the GABA and GLU neurotransmission in this brain region (1). However, it is clear that because of the various factors involved in the activity of neurotransmitter systems in different brain regions, various studies are required to determine the role of GABA receptors in GABA and GLU transmissions (34).

## Conclusion

Our results suggest that morphine influence the GABA and GLU transmission in the mPFC. Dopaminergic projections from mesocorticolimbic system to the mPFC could influence the metabolism and concentrations of the GABA and GLU in mPFC region. It is possible that understanding the details of this neural circuitry may provide further insight into the mechanisms underlying morphine dependence. Meanwhile, further studies are required to improve our understanding of the GABAergic and glutamatergic system in drug dependence. 
